# Exploring miRNA Profiles in Colon Cancer: A Focus on miR101-3p, miR106a-5p, and miR326

**DOI:** 10.3390/cancers16122285

**Published:** 2024-06-20

**Authors:** Constantin-Dan Tâlvan, Elena-Teodora Tâlvan, Călin Ilie Mohor, Liviuța Budișan, Valentin Grecu, Manuela Mihalache, Oana Zănoagă, Sergiu Chira, Ioana Berindan-Neagoe, Victor Cristea, Cosmin Ioan Mohor

**Affiliations:** 1 Faculty of Medicine, “Lucian Blaga” University of Sibiu, 550169 Sibiu, Romania; talvan_dan@yahoo.com (C.-D.T.); calin.mohor@ulbsibiu.ro (C.I.M.); manuela.mihalache@ulbsibiu.ro (M.M.); cosmin.mohor@ulbsibiu.ro (C.I.M.); 2 Research Center for Functional Genomic, Biomedicine and Translational Medicine, “Iuliu Hațieganu” University of Medicine and Pharmacy Cluj-Napoca, 400012 Cluj-Napoca, Romania; liviuta.petrisor@umfcluj.ro (L.B.); oana.zanoaga@umfcluj.ro (O.Z.); sergiu.chira@umfcluj.ro (S.C.); ioananeagoe29@gmail.com (I.B.-N.); victor_cristea@yahoo.com (V.C.); 3 Faculty of Engineering, “Lucian Blaga” University of Sibiu, 550025 Sibiu, Romania; valentin.grecu@ulbsibiu.ro

**Keywords:** miRNAs, colon cancer, staging, tissue, biomarkers

## Abstract

**Simple Summary:**

This paper investigates the expression of miR-101-3p, miR-106a-5p, and miR-326 in colon cancer tissues compared to adjacent healthy tissues. This study involves 40 colon cancer patients, whose tissue samples were analyzed using qRT-PCR. Results indicate that these miRNAs are overexpressed in adjacent healthy tissues but decrease in advanced cancer stages. This study suggests a strong correlation between miR-106a-5p and miR-326 with colon cancer severity. The findings propose that miRNA profiling could be useful for early diagnosis and prognosis in colon cancer management.

**Abstract:**

Early diagnosis and prognosis of cancer progression through biomarker profiling are crucial in managing colon cancer patients. Our research aimed to investigate the expression of miR-101-3p, miR-106a-5p, and miR-326 in tumor and adjacent healthy tissues of colon cancer patients and determine their potential diagnostic utility. This study included 40 patients divided into four groups according to the TNM staging classification. MiRNA expression was analyzed using qRT-PCR. The results showed that miR-101-3p, miR-106a-5p, and miR-326 are overexpressed in adjacent healthy tissues but decrease in advanced cancer stages. MiR-106a-5p and miR-326 are strongly correlated with colon cancer severity. These findings suggest that miRNA profiling could be useful for early diagnosis and prognosis in colon cancer management.

## 1. Introduction

Colorectal cancer (CRC) represents a significant health challenge, ranking as the third and fourth most common cause of cancer-related mortality in women and men, respectively [1]. Risk factors for CRC include advanced age, lifestyle choices such as smoking and alcohol consumption, chronic intestinal inflammation, the presence of polyps, and genetic predisposition [2]. Despite its high mortality rate, early detection of CRC can lead to effective treatment and a potential cure [3,4]. The TNM staging system and molecular biomarkers are crucial in the clinical assessment of CRC, influencing both treatment strategies and prognostic outcomes [5]. In this context, biomarker profiling emerges as a pivotal tool for the diagnosis and management of CRC [6].

MicroRNAs (miRNAs) are short, non-coding RNA molecules, typically 20–24 nucleotides in length, that play a regulatory role in gene expression without translating into proteins [7,8]. These miRNAs exert their influence by binding to messenger RNAs, thereby modulating various signaling pathways involved in carcinogenesis and cellular proliferation [9]. Aberrant miRNA expression is implicated in a range of disorders, including cancer, cardiovascular, bone, metabolic, and autoimmune diseases [10]. Specifically, dysregulated miRNA expression can initiate carcinogenesis and tumor progression, highlighting their potential as valuable biomarkers for cancer diagnosis and prognosis [11,12,13]. Overexpressed miRNAs may function as oncogenes, promoting tumor growth by affecting cell differentiation, growth, and apoptosis, while underexpressed miRNAs may act as tumor suppressors, inhibiting cell proliferation and invasion [10]. Thus, evaluating miRNA expression in colon tumor tissues could serve as a diagnostic and therapeutic response biomarker for individuals at high risk of CRC or those already diagnosed [3].

MiRNAs play crucial roles in various types of cancer. MiR-101-3p acts as a tumor suppressor in lung adenocarcinoma (LUAD) by sensitizing cells to radiotherapy through targeting BIRC5 [14]. In contrast, miR-106a-5p exhibits dual roles in different cancers: promoting laryngeal cancer (LC) development by activating the AKTIP/PI3K/AKT/m-TOR axis [15] but functioning as a tumor suppressor in clear cell renal cell carcinoma (ccRCC) by targeting VEGFA [16]. Additionally, exosomal miR-106a-5p contributes to chemoresistance in nasopharyngeal carcinoma (NPC) by targeting ARNT2 and promoting tumorigenesis [17]. Although miR-326 was not directly mentioned in the provided contexts, its potential roles in cancer remain an area for further exploration based on the diverse functions exhibited by different miRNAs in cancer progression and treatment resistance.

The expression of miR-101-3p, miR-106a-5p, and miR-326 can be influenced by various factors such as exposure to sulfur mustard, insulin resistance, and endometrial cancer. Studies have shown that sulfur mustard exposure affects the expression of miR-106a-5p and miR-106b-5p in peripheral blood mononuclear cells, potentially contributing to chronic pulmonary complications [15,18]. In the context of insulin resistance and nonalcoholic fatty liver disease, miR-101-3p is downregulated, promoting progressive NAFLD through hepatocyte and hepatic stellate cell interactions [18]. Additionally, in endometrial cancer, miR-101 downregulation is associated with increased mTOR expression, affecting cell proliferation, apoptosis, and invasion through the PI3K/Akt/mTOR signaling pathway [19]. These findings highlight the diverse influences of different commodities on the expression of these microRNAs, emphasizing their roles in various physiological and pathological processes.

MiRNAs are integral to CRC pathogenesis, influencing processes such as proliferation, angiogenesis, metastasis, and apoptosis, either as oncogenes or tumor suppressors [20]. MiR-101, which generates two precursors, miR-101-3p and miR-101-5p, is predominantly involved in regulating tumorigenesis and progression through its dual oncogenic and suppressive effects [21,22]. MiR-101-3p, the more stable form, has been identified as a tumor suppressor, is downregulated in various cancers, and is associated with inhibiting cell invasion and migration and promoting apoptosis in lung cancer [23,24,25,26,27,28]. Similarly, miR-106a-5p has been linked to CRC progression by targeting genes that inhibit tumor cell death and enhance cell migration and invasion [29]. Its expression varies across different cancers, acting as an oncogene in most except for renal carcinoma, glioma, and astrocytoma, where it is downregulated and associated with better survival outcomes [30,31,32]. MiR-326, recognized for its tumor-suppressive role, has been shown to inhibit cell proliferation and invasion in melanoma and endometrial cancer, while its low expression correlates with increased cell proliferation, migration, and lymph node metastasis in other cancers [33]. Elevated levels of miR-326 have been linked to reduced progression-free and overall survival in colorectal cancer patients, although its precise role in CRC remains to be fully elucidated [34,35,36,37].

Our study delves into the expression of miR-101-3p, miR-106a-5p, and miR-326 in both tumoral and adjacent non-tumoral tissues of CRC patients, seeking to establish correlations among these miRNAs across various stages of the disease.

## 2. Material and Method

### 2.1. Study Population

The present study encompassed a cohort of 40 patients diagnosed with colon cancer who underwent surgical procedures at the Regional Institute of Gastroenterology and Hepatology in Cluj-Napoca and the County Emergency Hospital of Sibiu within a span of 12 months. The patient population, comprising 22 females and 18 males with ages ranging from 45 to 86 years, was categorized into four distinct groups based on the TNM staging classification for colon and rectal cancer (see Table 1).

### 2.2. Sample Collection

This research involved a cohort of 40 individuals diagnosed with colon adenocarcinoma confirmed through histopathological examination. During colon cancer surgery, two tissue specimens were obtained from each patient: one from the tumor site and another from the surrounding peritumoral area, which were subsequently scrutinized for signs of microscopic cell infiltration. These tissue samples underwent specific preparation procedures and were then preserved in liquid nitrogen at a temperature of −170 °C for future analysis. Prior to their involvement in this study, every participant was given a detailed overview of the research objectives, and subsequent to a comprehensive medical assessment, they granted their informed consent to participate. The inclusion criteria necessitated that the patients have a confirmed diagnosis of colon adenocarcinoma through histopathology, possess a satisfactory performance status, and have not received any prior chemotherapy or radiotherapy treatments. In contrast, individuals were excluded from the study if they had autoimmune or inflammatory disorders, had undergone therapies that could impact miRNA expression, had other types of cancer, were experiencing active infections, or had encountered cardiovascular incidents within the preceding six months.

### 2.3. RNA Isolation and Extraction 

Total RNA was extracted and isolated from all 40 paired colon adenocarcinoma samples, including both tumor and adjacent tissues, utilizing TRIzol™ Reagent (Thermo Fisher Scientific, Waltham, MA, USA) in accordance with the manufacturer’s guidelines.

The quantification (concentration and quality) of RNA was performed by using a NanoDrop-1000 spectrophotometer (ThermoFischer Scientific, Waltham, MA, USA). After quantification, the samples were then diluted to 50 ng/μL for all the samples.

cDNA Synthesis and Quantitative Real-Time RT-PCR: miRNA expression levels were measured in 80 samples, encompassing both tumor and adjacent normal tissues. Following quantification, 50 ng of total RNA was reverse-transcribed into cDNA using a TaqMan miRNA Transcription Kit (ThermoFisher Scientific, Waltham, MA, USA) and a TaqMan miRNA Assay (ThermoFisher Scientific, Waltham, MA, USA) for the selected miRNAs (miR-101-3p, miR-106a-5p, and miR-326), following the manufacturer’s instructions. The stem-loop sequences for the selected miRNAs are presented in Table 2. 

The quantitative real-time polymerase chain reaction (qRT-PCR) was executed within a total volume of 10 μL, which included 5 μL of complementary DNA (diluted in a 1:5 ratio with nuclease-free water), 5.03 μL of TaqMan Fast Advanced Master Mix from Applied Biosystems (Waltham, MA, USA), and 0.47 μL of primer specific for each microRNA, utilizing the ViiA7 PCR system also from Applied Biosystems. The experimental parameters were established in the following manner: an initial denaturation phase at 50 °C for a duration of 2 min succeeded by 95 °C for 2 s then followed by 40 cycles comprising 95 °C for 1 s and 60 °C for 20 s. For normalization purposes, RNU48 and RNU6B were utilized as internal reference controls. RNU48 and RNU6B are commonly used in miRNA quantification due to their consistent expression levels, which is a critical attribute for reliable normalization. By employing both RNU48 and RNU6B, we aimed to mitigate the potential variability that might arise from using a single reference gene. The combined score of these snRNAs provides a more robust normalization factor, ensuring that the miRNA expression data are accurately calibrated and reflective of true biological differences rather than technical variations [38]. The ΔΔCT method was used for the analysis of the obtained CT values. GraphPad Prism software v.6 (GraphPad Software, San Diego, CA, USA) was used for statistical analysis of the obtained results. The qRT-PCR was performed in duplicates for each sample. 

### 2.4. Statistical Analysis

The research on miR101-3p, miR106a-5p, and miR-326 tissue expression in colon cancer involved the following statistical analysis. The data underwent initial organization and cleaning through the utilization of Microsoft Excel (Version 2405, Built 17628.20144), which was succeeded by the importation of the data into MiniTab 20 software for further detailed analysis. In order to gain a comprehensive understanding of the dataset, various descriptive statistics were computed, encompassing measures such as mean, standard deviation, minimum, maximum, and median, which were then arranged in tabular format for clear presentation. To visually explore the distribution and variability within the dataset, graphical techniques including boxplots and histograms were utilized, enabling the detection of any potential outliers or skewness that might impact the analysis. Moving beyond descriptive statistics, a multiple regression analysis was executed to delve into the intricate relationship between the microRNAs and the status of colon cancer while accounting for covariates such as age, gender, and disease stage to control for potential confounding factors. The results of the regression analysis were then examined, with a detailed presentation of regression coefficients, standard errors, *p*-values, and R-squared values, allowing for a comprehensive interpretation of the findings. Furthermore, in order to compare the average values of microRNAs between adjacent peritumoral tissue and cancer tissue, two-sample *t*-tests were conducted under the assumption of equal variances to assess the statistical significance of the differences observed. The outcomes of these t-tests were rigorously analyzed, with the reporting and interpretation of T-statistics, degrees of freedom, *p*-values, and confidence intervals, all while maintaining a predetermined significance level of 0.05 for all statistical tests conducted. miRDB web software PMID: 31504780 was used for target genes prediction of the three miRNAs. The online tool ShinyGO version 0.76.3 PMID: 31882993 was used to generate charts for functional and pathway enrichment analysis. 

## 3. Results

### 3.1. Demographic Analysis

Age distribution by colon cancer staging indicates that most patients were diagnosed at stage 2 or 3. Notably, the mean age was lower in stages 1 and 3 and higher in stages 2 and 4. Advanced stages of colon cancer tend to be diagnosed in older patients (see Table 3). Additionally, male patients were generally older than females across most stages, except for stage 2. The observed phenomenon where male patients diagnosed with stage 2 colon cancer are younger than their female counterparts, while in all other stages men are older, may be attributed to several factors such as hormonal differences between sexes, lifestyle factors such as diet, smoking, and alcohol consumption, which differ statistically between genders, or screening practices and healthcare access that can influence the age of diagnosis. It is important to note that these are hypotheses that would require further investigation to substantiate.

### 3.2. Descriptive Analysis of miRNA Expression

Table 4 presents a detailed analysis of the expression levels of three microRNAs (miR 101-3p, miR 106a-5p, and miR326) across different age groups for both female and male patients. The data are broken down by age cohorts spanning from 40 to 89 years, with the number of patients (N) in each group varying.

**Figure 1:**
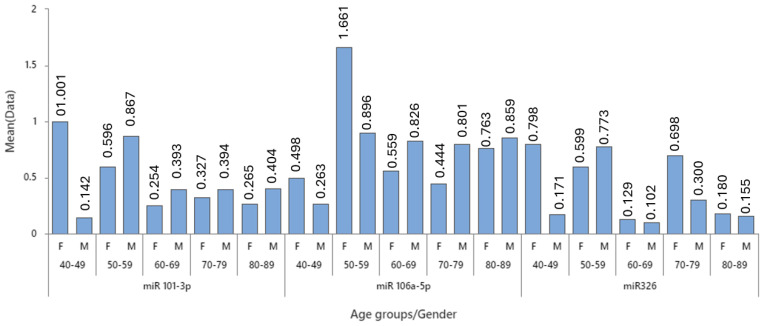
Expression of MiR-101-3p, MiR-106a-5p, and MiR-326 between females and males in different age groups: Female 40–49 (n = 2); 50–59 (n = 7); 60–69 (n =5); 70–79 (n = 4); 80–89 (n = 4). Male: 40–49 (n = 1); 50–59 (n = 1); 60–69 (n − 8); 70–79 (n = 4); 80–89 (n = 4).

For miR 101-3p, the highest mean expression in females is observed in the 40–49 age group, while for males, it peaks in the 50–59 age group, followed by a subsequent decline in older cohorts. Notably, the overall expression of miR-101-3p was marginally higher in male patients compared to female patients, apart from the 40–49 age group where a pronounced overexpression was observed in females. This trend suggests that young female patients with colon cancer may exhibit increased levels of miR-101-3p, as depicted in Table 3 and Figure 1. The standard deviation (StDev) and variance indicate the spread and dispersion of expression levels within these groups, with larger values suggesting greater variability. 

In the case of miR 106a-5p, females in the 50–59 age group show a notably high mean expression, but this is based on a small sample size, which could be skewed by outliers, as indicated by the high standard deviation. For males, a more consistent expression level is seen across older age groups, who show higher levels of miR-106a-5p expression compared to their female counterparts, as depicted in Table 3 and in Figure 1.

For miR326, the expression levels are relatively lower compared to the other miRNAs, with females in the 70–79 age group showing the highest mean expression. The analysis indicated that miR-326 tends to be expressed at higher levels in female patients across most age groups, with the exception of the 50–59 age group. This trend was especially evident in the 40–49 and 70–79 age cohorts (see Figure 1). Overall, miR-101-3p and miR-106a-5p exhibited a greater expression in male patients, whereas miR-326 levels were predominantly higher in female patients. 

Figure 1 highlights potential age- and gender-related differences in miRNA expression in colon cancer patients, providing a better view of the results presented in Table 3.

**Figure 2:**
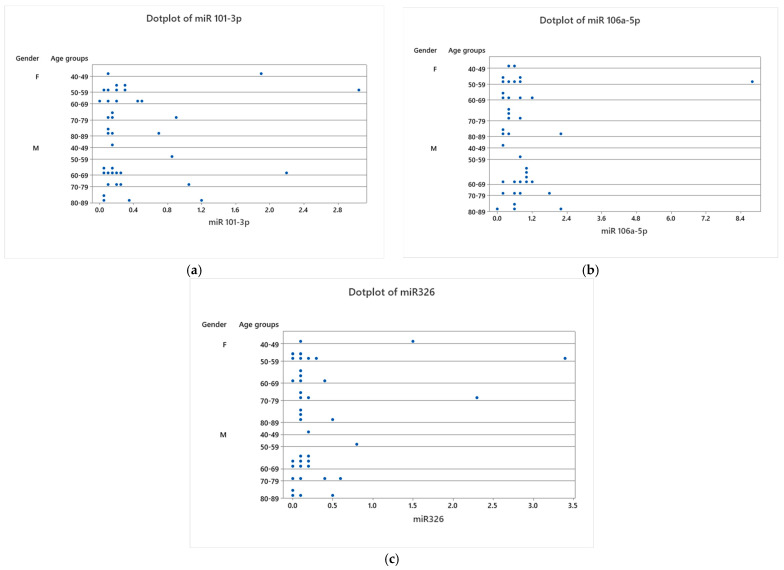
Dotplots for the expression of MiR–101–3p (**a**), MiR–106a–5p (**b**), and MiR–326 (**c**) between females and males in different age groups in tumoral tissues.

Figure 2 offers a detailed comparison of miR-101-3p, miR-106a-5p, and miR-326 expression levels across various age groups and between genders within cancer tissues. This figure elucidates the process behind the calculation of mean values depicted in Table 3 and Figure 1 by displaying the expression data for each patient analyzed. Table 3 and Figure 2 reveal the presence of outliers, particularly within the 50–59 female age group, which influence the average expression values of the miRNAs. 

**Figure 3:**
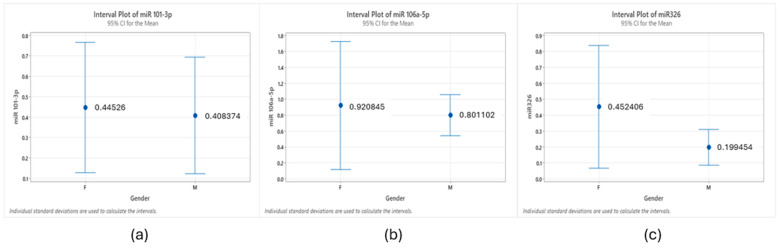
Mean expression of miRNAs by gender: (**a**) miR-101, (**b**) miR106, and (**c**) miR-326. Female (n = 22); male (n = 18).

Figure 3 delineates the differential expression patterns of the three miRNAs, highlighting a gender-based disparity. Notably, miR-106a-5p and miR-326 exhibit an upregulation in female patients when contrasted with male counterparts. The expression patterns observed may suggest a potential for gender-specific roles, which warrants further investigation.

**Figure 4:**
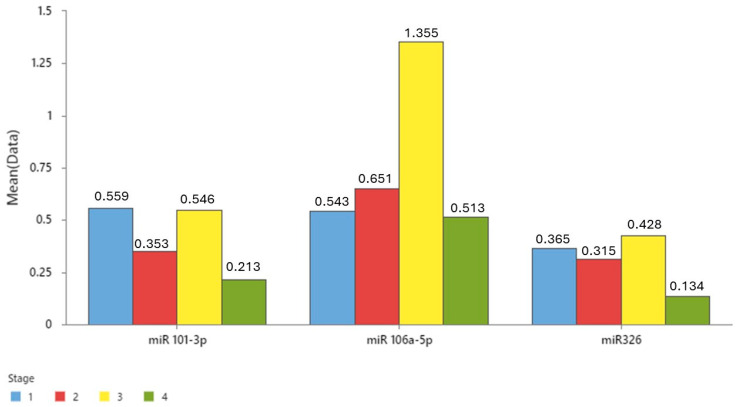
MiRNA expression in tumoral tissue in different stages of colon cancer: stage 1 (n = 5); stage 2 (n = 16); stage 3 (n = 14); stage 4 (n = 5).

MiRNAs analysis in tumoral tissue between different stages of colon cancer, presented in Figure 4, shows an enhanced expression of all three miRNAs in stage 3 colon cancer (especially of miR-106a-5p). The expression of all three miRNAs appears to be elevated in the early stages of colon cancer, with a notable increase observed in stage 3, although this observation warrants further investigation due to the limited sample size in stage 4.

**Figure 5:**
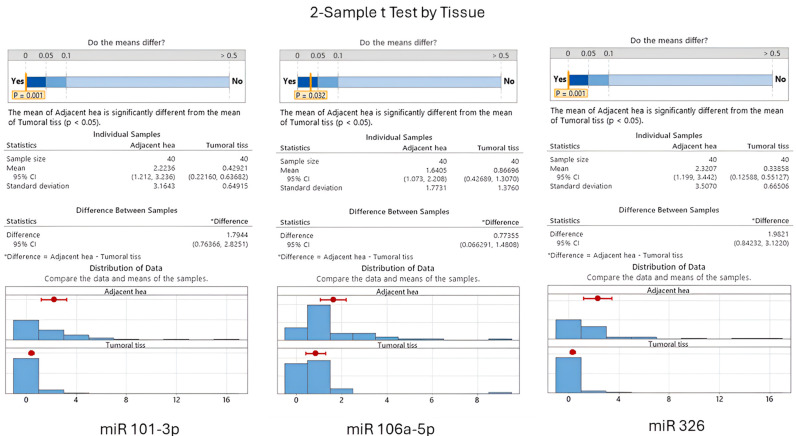
Comparison of miRNAs in adjacent peritumoral tissue and cancer tissue for miR-101, miR106, and miR-326 (visual representation generated with MiniTab 20).

The comparative analysis of miRNA expression in cancerous and peritumoral tissues revealed a lateral asymmetry, as shown in Figure 5.

The *p*-values obtained from the two-sample *t*-tests—(*p* = 0.001) for miR-101-3p, (*p* = 0.032) for miR-106a-5p, and (*p* = 0.001) for miR-326—underscore the robustness of the observed differences in expression. It was discerned that the mean expression levels of these miRNAs were consistently higher in healthy tissue as opposed to tumoral tissue. This pronounced expression in non-cancerous tissue suggests a potential inverse relationship with tumorigenesis.

### 3.3. Correlations between miRNAs in Cancer and Peritumoral Tissues

**Figure 6:**
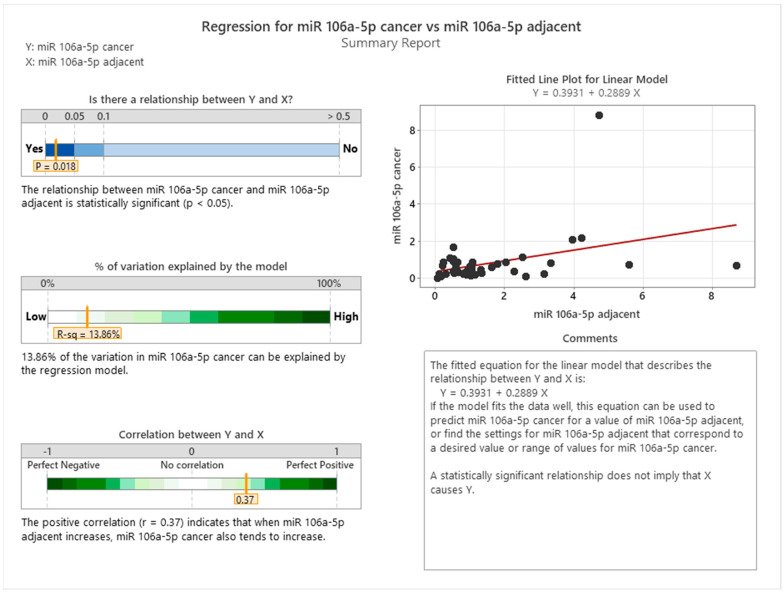
Regression between adjacent peritumoral tissue and cancer tissue for miR−106a−5p (visual representation generated with MiniTab 20).

We explored the relationship between the expression levels of miR-106a-5p in healthy peritumoral tissues and cancerous tissues, as illustrated in Figure 6. Regression analysis revealed a statistically significant, yet modest, linear correlation between the expression of miR-106a-5p in healthy peritumoral tissue and tumoral tissue. The correlation coefficient suggests that approximately 13.86% of the variance in miRNA expression in cancerous tissue can be accounted for by its expression in healthy tissue. This finding gently challenges the prevailing assumption that the expression of tumor-suppressor microRNA is unaffected by adjacent healthy tissue, proposing a potential predictive relationship between the two.

The expression levels of miR-106a-5p in the peritumoral region may serve as a preliminary indicator of its levels within the tumor, which could imply the existence of a systemic regulatory mechanism or a field effect in carcinogenesis. This observation tentatively supports the notion that there may be consistent miRNA expression profiles in individual patients across different tissue types. Our analysis contributes an initial insight into the complex interplay between tumoral and adjacent non-tumoral tissues, potentially enriching the current understanding of tumor biology and informing the development of diagnostic approaches. The use of healthy tissue expression as an independent variable in our regression model is tentatively supported by these results, which may enhance the clinical relevance of our findings.

### 3.4. Correlations between miRNAs in Cancer Patients

**Figure 7:**
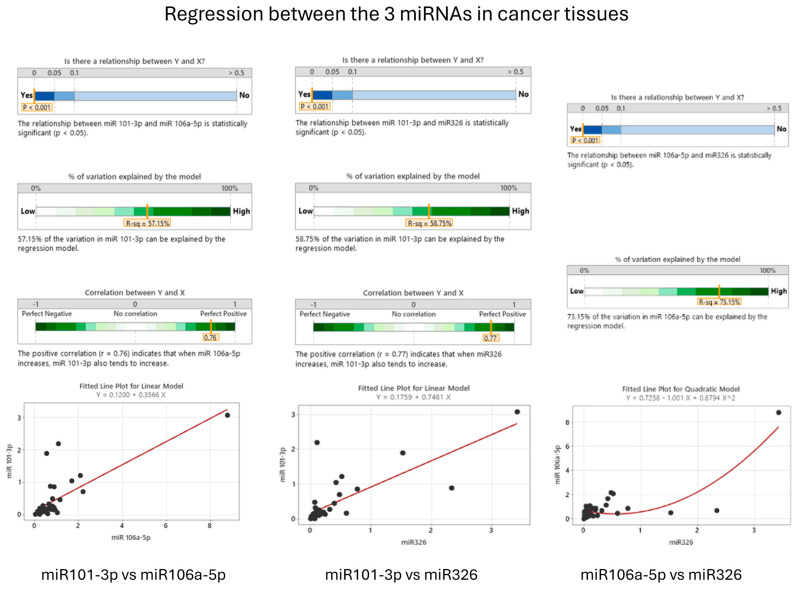
Correlation between miR−101−3p, miR−106a−5p, and miR−326 in cancer tissues (visual representation generated with MiniTab 20).

Our analysis identified a statistically significant correlation between miR-101-3p and miR-106a-5p in tumoral tissues (*p* < 0.001). This finding suggests a possible interplay between the expression patterns of miR-101-3p and the behavior of miR-106a-5p in malignant tissues. The observed association, as presented in Figure 7, may indicate a synergistic or interdependent role in the molecular processes of colon cancer. Additionally, a significant correlation was also noted between miR-101-3p and miR-326 in cancerous tissues (*p* < 0.001), hinting at parallel trends in the expression of these microRNAs and raising the possibility that miR-326 may influence the expression of miR-101-3p in colon cancer cases.

These preliminary observations suggest that further research is needed to understand the complex interactions and potential regulatory functions of miR-101-3p, miR-106a-5p, and miR-326 in the progression and treatment of colon cancer. Such insights are crucial for elucidating the molecular mechanisms at play and could inform therapeutic strategies. Future investigations could focus on the functional consequences of these miRNA relationships and their role in cancer development, aiming to uncover new pathways for tailored medical treatments for colon cancer patients.

Correlations between miR-106a-5p and miR-326 also show an important interdependence between these two miRNAs in cancer tissues (*p* < 0.001). MiR-326 can strongly influence expression of miR-106a-5p in patients with colon cancer, as shown in Figure 7.

The interplay between miR-106a-5p and miR-326 within colon cancer tissues has been substantiated by a statistically significant correlation, as evidenced by a *p*-value of less than 0.001. This compelling association suggests a mutual influence on the expression levels of these miRNAs in the oncogenic context. The regression analysis, as depicted in Figure 6, further corroborates this interdependence with a fitted line plot for a quadratic model, which delineates a clear trajectory of their relationship.

The model elucidates that approximately 73.15% of the variation in miR-106a-5p expression can be accounted for by the regression relative to miR-326. This high percentage of explained variation signifies a strong predictive capacity of the model. The quadratic equation (Y = 0.7258 − 1.001X + 0.8794X^2) derived from the analysis provides a mathematical framework for predicting the expression levels of miR-106a-5p based on miR-326 values, although it does not imply causality.

**Figure 8:**
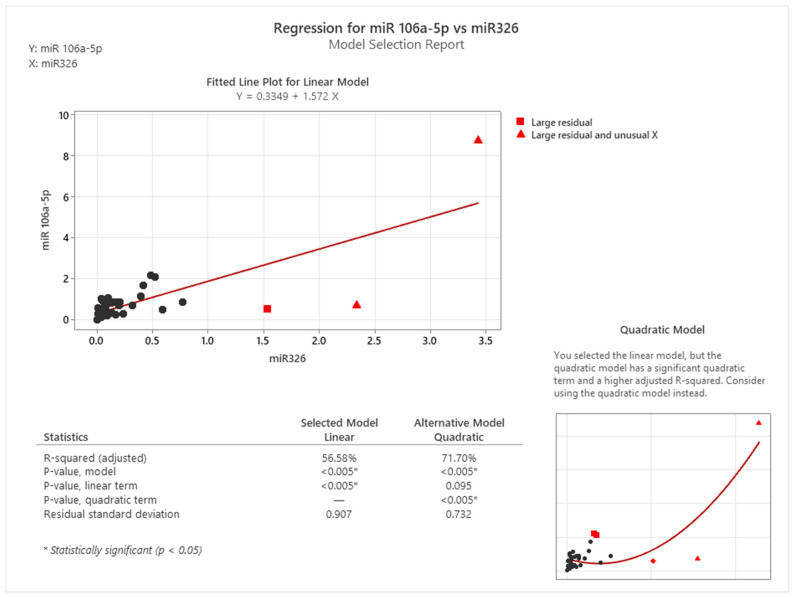
Correlation between miR-106a-5p and miR-326 in cancer tissues: linear versus quadratic (visual representation generated with MiniTab 20).

As observed in Figure 7, there is a linear correlation between miR-101-3p and miR-106a-5p and between miR-101-3p and miR-326, while the relationship between miR-106a-5p and miR-326 is presented using a quadratic equation. Figure 8 provides a compelling justification for this choice.

The comparative assessment between linear and quadratic regression models for the relationship between miR-106a-5p and miR-326 demonstrates that the quadratic model offers a more accurate fit. This is evidenced by a higher adjusted R-squared value of 71.70% and a significant *p*-value for the quadratic term, both indicative of the model’s robustness in capturing the nuanced relationship between these two miRNAs. The linear model, while initially considered, showed large residuals and unusual X values, leading to a less reliable fit with an adjusted R-squared value of 56.58%. The quadratic model’s superior fit is further supported by the significant quadratic term, suggesting that the relationship between miR-106a-5p and miR-326 is not strictly linear but rather follows a curved trajectory. This analysis substantiates our decision to employ a quadratic equation in Figure 6, distinguishing it from other correlations where linear models were sufficient and appropriate. The quadratic model’s enhanced descriptive power underscores its suitability for capturing the complex dynamics of miRNA interactions in colon cancer progression.

The utilization of a quadratic model to describe the relationship between miR-106a-5p and miR-326 is further justified by the biological intricacies of miRNA interactions. In biological systems, interactions between molecules such as miRNAs are rarely linear; they are often influenced by a multitude of factors that can lead to synergistic or antagonistic effects. The quadratic model is more adept at capturing these complex interactions, which may involve feedback loops or regulatory networks that are not apparent in a linear framework. This is particularly relevant in the context of cancer progression, where miRNA interactions can have profound implications on cellular pathways and disease outcomes. The quadratic equation, therefore, provides a more nuanced and accurate representation of the miRNA relationship, aligning with the biological reality of their interaction within the cellular milieu.

### 3.5. Correlations between miRNA in Cancer Staging

**Figure 9:**
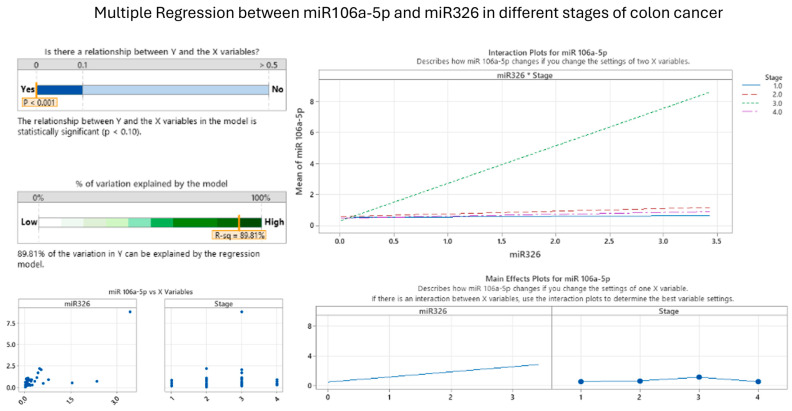
Correlation between miR-106a-5p and miR-326 in different stages of colon cancer (visual representation generated with MiniTab 20).

The comprehensive analysis of miR-106a-5p and miR-326 across the four stages of colon cancer revealed a statistically significant correlation, suggesting a parallel trend in their expression during the progression of the disease, as shown in Figure 9. This relationship is crucial for understanding the dynamics of colon cancer development.

Figure 9 depicts an interaction plot showcasing the consistent rise in the average expression levels of miR-106a-5p as colon cancer advances through its various stages, which are distinguished by different colors assigned to each stage. The visual representation clearly shows a direct relationship between the mean expression of miR-106a-5p and the levels of miR-326, suggesting that as the disease progresses from the initial stage to the advanced stage, there is a noticeable increase in both microRNAs, with the strongest association observed at stage 3. This pattern underscores the significance of these specific molecules as potential biomarkers intricately associated with the seriousness and progression of colon cancer.

### 3.6. Functional Enrichment Analysis of miRNAs’ Target Genes

To highlight the cellular pathways that are regulated by the three miRNAs (miR-101-3p, miR-106a-5p, and miR-326), we performed target gene prediction analysis. Gene sets potentially targeted by the three miRNAs were further analyzed with ShinyGO software for pathway enrichment, using the KEGG analysis module. The common signaling pathways regulated by the three miRNAs are PI3K-Akt and MAPK, among others that are involved in carcinogenesis. 

**Figure 10:**
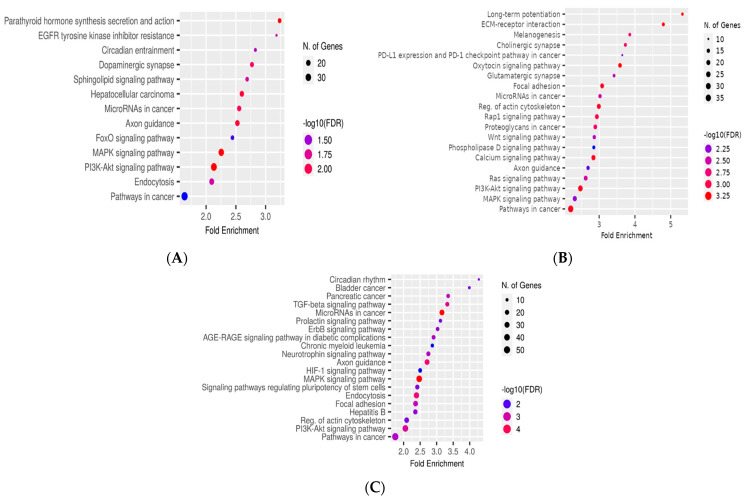
Gene set enrichment analysis for (**A**) miR−101−3p, (**B**) miR−106a−5p, and (**C**) miR−326 (visual representation generated with ShinyGO version 0.76.3 PMID: 31882993).

Figure 10, encompassing parts A, B, and C, provides a gene set enrichment analysis for miR-101-3p, miR-106a-5p, and miR-326, each part corresponding to one of the microRNAs. The analysis highlights the significant association of these miRNAs with various pathways involved in cancer and cellular signaling. The common feature across all three parts of Figure 10 is the involvement of the selected miRNAs in regulating pathways that are critical in carcinogenesis, such as the PI3K-Akt and MAPK pathways. These findings justified the rationale for choosing these three miRNAs for our study.

## 4. Discussion

Expression levels of miRNAs can be important diagnostic markers for cancer and can help a clinician in assessing the risk of a patient of developing cancer or in tumor staging. It allows analysis from small biopsies and can be extracted from body fluids through non-invasive procedures [39]. The main purpose of our study was to assess tumoral and peritumoral tissue expression of miR-101-3p, miR-106a-5p, and miR-326 in colon cancer patients and to correlate their expression with tumor staging in order to facilitate their future use as diagnostic tools. 

The incidence of colon cancer in the young population is rising, and as a result, guidelines suggest beginning screening from the age of 45 [40]. Auxiliary diagnostic methods such as miRNA expression and behavior could be useful in early detection and prognosis of this malignancy [13,41]. Descriptive analysis in this study showed associations between different stages of colon cancer, age, gender, and miRNA expression. Age distribution in our study showed that diagnosis incidence was at its highest in stages 2 and 3 of colon cancer and that the mean age of patients was lower in stages 1 and 3 of colon cancer (64 years). Studies show that over 80% of cancer patients are >50 years old at the time of diagnosis and observe a concerning increase in this malignancy in young patients (between 40 and 50 years of age) and lower incidence in older patients (>85 years of age) [42]. Nearly 90% of patients are diagnosed after already experiencing symptoms or have blood and radiological alterations [43]. However, the majority of colorectal cancer patients are diagnosed in a local stage, and under 10% are diagnosed in an advanced stage [44]. Age and gender distribution in colon cancer staging in our study shows that male cancer patients are slightly older than women in most stages (except stage 2). Some studies pointed out that women with colon cancer were older and showed more regional disease than men [45] but had higher grading and stage at diagnosis of primary tumor [46]. 

Transcriptome profiling connects cellular behaviors and their mutation and can reveal new biomarkers and treatment options in colon cancer [12]. We found that miR-101-3p and miR-106a-5p are elevated in younger cancer patients and decline with older age and are slightly enhanced in men compared to women between different age groups. Studies show a downregulation of miR-101-3p in frailty syndrome, an age-related pathology [47]; however, we found no studies regarding expression differences of these miRNAs between genders in cancer patients. MiR-326 was shown to have a slightly higher expression in females than males with colon cancer in most age groups compared to the other two miRNAs in our study. However, all three miRNAs were found to have an enhanced expression in younger age groups of colon cancer patients. Overall, in this study, all three miRNAs were slightly enhanced in women compared to men, suggesting that gender does not have an impact in their expression in colon cancer patients. However, estrogen has been shown to be implicated in the differential regulation of miRNA expression, which may elucidate the slightly heightened levels of miR-326 in female subjects [48,49,50].

Regarding tissue expression of miR-101-3p, miR-106a-5p, and miR-326, we found that all three miRNAs are overexpressed in the adjacent non-tumor tissues compared to cancer tissues. The expression profiles of miR-101-3p, miR-106a-5p, and miR-326 demonstrate a marked elevation in peritumoral tissues relative to tumoral counterparts. This is particularly notable for miR-101-3p and miR-326. The significantly diminished expression of miR-101-3p and miR-326 in tumoral tissues may indicate an inverse correlation between their levels and the presence of cancer, suggesting their potential role in tumorigenesis. Other studies show a suppression of miR-101 expression in cancer tissues [51,52] similar to our study but a shortened overall survival in patients with enhanced miR-106 [53,54] and high expression of miR-326 in patients with decreased progression-free survival [34]. Moreover, we demonstrated that between different stages of colon cancer, all three miRNAs showed decreased expression in advanced stages of colon cancer compared to earlier stages. 

Two-sample *t*-test analysis between miRNA expression in tumoral and peritumoral tissues shows different results. The differential expression of three pivotal miRNAs—miR-101-3p, miR-106a-5p, and miR-326—was rigorously quantified in healthy peritumoral tissue compared to tumoral tissue derived from colon cancer patients. Employing two-sample t-tests, this study sought to elucidate the variance in expression levels between these distinct tissue types. The ensuing results revealed significant statistical disparities, thereby substantiating the hypothesis that the mean expression of the miRNAs is not uniform across healthy and cancerous tissues. The implications of these findings are multifaceted. Firstly, the heightened expression of miR-101-3p, miR-106a-5p, and miR-326 in healthy tissue may indicate a protective or regulatory role that is compromised in the cancerous state. Secondly, the significant decrease in expression within tumoral tissues posits these miRNAs as potential biomarkers for the presence and progression of colon cancer. Lastly, the strong statistical evidence provided by the low *p*-values reinforces the potential clinical relevance of these miRNAs in the diagnostic landscape of colon cancer.

This analysis suggests that the expression profiles of miR-101-3p, miR-106a-5p, and miR-326 may hold critical insights into the molecular mechanisms underpinning colon cancer development and could serve as valuable indicators in the disease’s early detection and prognosis. 

This study examined the correlation between the expression levels of miR-101-3p in healthy peritumoral tissues and their tumoral counterparts. Statistical analysis employing Pearson’s correlation coefficient revealed no significant association between the two tissue types (*p* = 0.278), (*p* > 0.05). This lack of statistical significance suggests that the expression of miR-101-3p in healthy adjacent tissue does not serve as a reliable predictor for its expression in tumoral tissues. Furthermore, the findings imply that the regulatory mechanisms governing miR-101-3p expression in healthy peritumoral tissues are distinct from those in cancerous tissues. This independence indicates that miR-101-3p may participate in different biological pathways depending on the tissue context, which could have implications for its role in tumorigenesis and potential utility as a biomarker. The absence of a predictive relationship also underscores the complexity of miRNA regulation within the tumor microenvironment. It suggests that while miR-101-3p expression is altered in the presence of cancer, it is not directly influenced by the levels found in the surrounding healthy tissue. This observation could guide future research into the specific functions and interactions of miR-101-3p within the cellular milieu of colon cancer. However, miR-106a-5p expression in healthy tissues can predict its expression in tumoral tissues, the relationship between them being statistically significant. This finding suggests that miR-106a-5p could serve as a valuable indicator in the assessment of tumor biology and the progression of colon cancer, offering insights into the underlying molecular mechanisms of the disease. Investigation into the correlation of miR-326 expression between cancerous and peritumoral tissues yielded no significant relationship (*p* = 0.8), (*p* > 0.05). This indicates that the levels of miR-326 in peritumoral tissues do not exert an influence on its expression within cancer tissues. The absence of correlation suggests independent regulatory mechanisms for miR-326 in these distinct tissue environments, which may have implications for its role in the pathophysiology of colon cancer. We could not find any studies to describe the correlation between these miRNAs in tumoral and peritumoral tissues in cancer patients.

We also conducted an analysis between the expression of all three miRNAs in cancer tissues and observed that miR-101-3p and miR-106-5p were positively correlated and can influence each other’s behavior (*p* < 0.001). We also showed that miR-101-3p is positively correlated with miR-326 and that there is also a strong interdependence between miR-106a-5p and miR-326 (*p* < 0.001), showing that they can influence each other’s expression in cancer tissues. This analysis not only highlights the significant interdependence between miR-106a-5p and miR-326 in cancerous tissues but also presents a quantitative approach to understanding their co-regulation in the pathogenesis of colon cancer. The findings may pave the way for further research into the functional implications of these miRNAs and their potential as biomarkers for diagnostic and prognostic purposes in colon cancer management. 

Moreover, the correlation between miRNAs in colon cancer staging showed interesting results. Mir-101-3p and miR-106a-5p cannot be positively correlated with colon cancer staging; also, the association between miR-101-3p and miR-326 in different stages of colon cancer is not statistically significant. We showed a statistically significant correlation between miR-101-3p and miR-106a-5p in the context of colon cancer (*p* < 0.001), but despite this strong correlation, the expression patterns of these miRNAs do not align consistently across the four stages of colon cancer. This inconsistency suggests that while miR-101-3p and miR-106a-5p are related, their relationship does not translate into a positive correlation with the staging of colon cancer. Consequently, the correlation between these two miRNAs is not indicative of the progression of the disease. Their association is not relevant in colon cancer staging, and these miRNAs cannot influence each other between different stages of colon cancer. However, the correlation between miR-106a-5p and miR-326 in different stages of colon cancer shows that these two are strongly associated and have the same behavior in cancer progression. The significant correlation between miR-106a-5p and miR-326, coupled with their consistent behavior across all stages, underscores their potential role not only as indicators but also possibly as contributors to the disease’s development or progression. Cancer stage has a much lower impact on the values of miR-106a-5p than the variation in miR-326. The findings from our study emphasize the importance of these miRNAs in the molecular pathology of colon cancer and their potential utility in clinical applications for diagnosis and prognosis.

Our quantitative real-time polymerase chain reaction (qPCR) analysis demonstrates that miR-101-3p, miR-106a-5p, and miR-326 exhibit decreased expression levels in the later stages of colon cancer, indicating a potential circumvention of the normal regulatory mechanisms governing oncogenic pathways, specifically the PI3K-Akt and MAPK pathways, which are crucial for tumor progression. The comprehensive examination of gene sets through enrichment analysis further emphasizes the significant role these microRNAs play in the process of carcinogenesis and highlights their promise as potential biomarkers for monitoring cancer advancement, given their ability to influence key pathways responsible for cell viability, growth, and specialization. The detailed results presented in Figure 10 shed light on the complex molecular interactions orchestrated by these miRNAs, proposing a substantial involvement in shaping cancer-related characteristics and providing valuable insights into disease staging and the identification of viable therapeutic targets. The observed decrease in the levels of these specific miRNAs could potentially enhance the aggressiveness of colon cancer, underscoring their critical functions in orchestrating the molecular events underlying cancer progression and emphasizing the broader implications of miRNA dysregulation in the pathophysiology of colon cancer.

## 5. Study Limitations

It is important to note that this research has several constraints that need to be recognized. First, regarding the sample size, the present study included only 40 patients, which is a relatively small sample size. This could limit the statistical power of this study and the generalizability of the results. The results showed that all three miRNAs tend to be enhanced in younger female patients. The miRNA panel is limited, as this study investigated only three miRNAs (miR-101-3p, miR-106a-5p, and miR-326). There are many other miRNAs that could potentially be relevant to colon cancer progression. There is a lack of longitudinal data, as this study is cross-sectional, analyzing the miRNA expression at a single point in time. A longitudinal study design, tracking changes in miRNA expression over time, might provide more insights into their role in cancer progression. This study divided patients into groups according to the TNM staging classification. However, this classification system has its own limitations and may not fully capture the complexity and heterogeneity of colon cancer. While this study found a strong interdependence between the three miRNAs in cancer tissues and a link between miR106a-5p and miR-326 and the severity of colon cancer, these are correlational findings. Further research is needed to establish a causal relationship. This also could suggest that other factors like associated comorbidities or lifestyle could influence the progression of the disease. This study concludes that increased numbers of patients and miRNAs in the panel are needed to determine the clinical utility of these biomarkers in colon cancer, suggesting that the current results may not yet be applicable in a clinical setting. These limitations should be considered when interpreting the results of this study. Further research is needed to confirm these findings and explore their implications.

## 6. Conclusions

This study aimed to investigate the expression of three miRNAs, miR-101-3p, miR-106a-5p, and miR-326, in tumoral tissue and adjacent peritumoral healthy tissue of patients with colon cancer and to establish correlations between them in different stages of colon cancer. Analysis of the patients included in this study revealed that the majority were diagnosed with stages two and three colon cancer. Across the entire cohort, it was observed that men tended to be slightly older than women, with the exception of stage 2, where male patients were younger than their female counterparts. Regarding miRNA expression in tumoral and healthy adjacent tissue in different age groups and genders, our findings show that miR-101-3p and miR-106a-5p were significantly increased in young women with colon cancer, but along with advancing age, they tend to be more expressed in males. MiR-326’s expression is evidently higher in females in most age groups. One of our main findings is that all the miRNAs show enhanced expression in adjacent peritumoral tissues compared to cancer tissues of patients with this malignancy; moreover, all three miRNAs show decreased expression in advanced stages of colon cancer. Another important finding is a significant positive correlation between all three miRNAs in cancer tissues; they have similar behavior and can influence each other. Also, the robust correlation observed between miR-106a-5p and miR-326 across various stages of colon cancer highlights their strong association and consistent behavior in disease progression. These miRNAs not only serve as potential indicators but may also play a contributory role in the development and advancement of the condition. 

The qPCR analysis indicates that the downregulation of the analyzed miRNAs may disrupt key regulatory pathways, notably PI3K-Akt and MAPK, enhancing tumor aggressiveness. These microRNAs emerge as potential biomarkers, with their target gene enrichment analysis revealing their influence on cell viability and proliferation.

In light of these insights, future research should aim to expand the miRNA panel and include a larger patient cohort to validate the specific roles of these miRNAs in colon cancer progression. Such studies could pave the way for the development of novel diagnostic tools and targeted therapies, potentially revolutionizing the management of colon cancer by enabling earlier detection and more personalized treatment approaches. Ultimately, the integration of miRNA expression profiles with clinical parameters may offer a more nuanced understanding of cancer biology and patient prognosis.

## Figures and Tables

**Table 1:** Clinicopathological characteristics of subjects.

1. Mean age (years)		67.9 (11.52) ^1^
2. Gender	Male	18 (45%)
	Female	22 (55%)
3. Tumor histology	Adenocarcinoma	40 (100%)
4. Tumor TNM stage	1	5 (12.5%)
	2	16 (40%)
	3	14 (35%)
	4	5 (12.5%)
5. Tumor WHO grade	1	9 (22.5%)
	2	24 (60%)
	3	7 (17.5%)
6. Tumor location	Right colon	21 (52.5%)
	Left colon	19 (47.5%)

^1^-Mean (standard deviation); WHO—World Health Organization.

**Table 2:** The stem-loop sequences for the selected miRNAs.

No.	miRNAs	Assay ID	Stem-Loop Sequence
1	RNU48	001006	GATGACCCCAGGTAACTCTGAGTGTGTCGCTGATGCCATCACCGCAGCGCTCTGACC
2	RNU6B	001093	CGCAAGGATGACACGCAAATTCGTGAAGCGTTCCATATTTTT
3	hsa-miR-101-3p	002253	UGCCCUGGCUCAGUUAUCACAGUGCUGAUGCUGUCUAUUCUAAAGGUACAGUACUGUGAUAACUGAAGGAUGGCA
4	hsa-miR-106a-5p	002169	CCUUGGCCAUGUAAAAGUGCUUACAGUGCAGGUAGCUUUUUGAGAUCUACUGCAAUGUAAGCACUUCUUACAUUACCAUGG
5	hsa-miR-326	000542	CUCAUCUGUCUGUUGGGCUGGAGGCAGGGCCUUUGUGAAGGCGGGUGGUGCUCAGAUCGCCUCUGGGCCCUUCCUCCAGCCCCGAGGCGGAUUCA

**Table 3:** Age and gender distribution in colon cancer staging.

Stage	Variable	Gender	N	Mean	SE Mean	StDev	Minimum	Q1	Median	Q3	Maximum
1	Age	F	3	59.67	6.36	11.02	47.00	47.00	65.00	67.00	67.00
		M	2	72.50	5.50	7.78	67.00	*	72.50	*	78.00
2	Age	F	8	73.88	4.21	11.90	56.00	60.00	78.00	84.00	85.00
		M	8	66.13	4.02	11.38	45.00	60.50	65.00	76.25	81.00
Stage	Variable	Gender	N	Mean	SE Mean	StDev	Minimum	Q1	Median	Q3	Maximum
3	Age	F	8	59.13	3.10	8.77	48.00	51.50	58.50	64.75	75.00
		M	6	72.50	3.85	9.44	59.00	65.00	72.00	82.25	83.00
Stage	Variable	Gender	N	Mean	SE Mean	StDev	Minimum	Q1	Median	Q3	Maximum
4	Age	F	3	72.00	8.33	14.42	56.00	56.00	76.00	84.00	84.00
		M	2	74.0	12.0	17.0	62.0	*	74.0	*	86.0

**Table 4:** Mean expression of the 3 miRNAs across age groups and genders.

Variable		Gender	N	Mean	SE Mean	StDev	Variance	Minimum	Q1	Median	Q3	Maximum
miR 101-3p	40–49	F	2	1.001	0.903	1.277	1.630	0.099	*	1.001	*	1.904
		M	1	0.14246	*	*	*	0.14246	*	0.14246	*	0.14246
	50–59	F	7	0.596	0.414	1.095	1.199	0.063	0.110	0.182	0.289	3.073
		M	1	0.86738	*	*	*	0.86738	*	0.86738	*	0.86738
	60–69	F	5	0.2547	0.0968	0.2165	0.0469	0.0234	0.0532	0.2066	0.4802	0.4955
		M	8	0.393	0.259	0.732	0.536	0.047	0.059	0.160	0.230	2.196
	70–79	F	4	0.327	0.188	0.376	0.141	0.118	0.125	0.150	0.706	0.891
		M	4	0.395	0.222	0.444	0.197	0.080	0.105	0.224	0.855	1.051
	80–89	F	4	0.265	0.149	0.299	0.089	0.097	0.100	0.126	0.570	0.712
		M	4	0.404	0.281	0.561	0.315	0.026	0.030	0.186	0.997	1.219
miR 106a-5p	40–49	F	2	0.4989	0.0550	0.0778	0.0060	0.4439	*	0.4989	*	0.5539
		M	1	0.26378	*	*	*	0.26378	*	0.26378	*	0.26378
	50–59	F	7	1.66	1.19	3.15	9.94	0.15	0.19	0.64	0.75	8.79
		M	1	0.89600	*	*	*	0.89600	*	0.89600	*	0.89600
	60–69	F	5	0.559	0.188	0.420	0.176	0.239	0.243	0.317	0.997	1.174
		M	8	0.827	0.103	0.291	0.085	0.216	0.660	0.908	1.032	1.108
	70–79	F	4	0.4448	0.0949	0.1897	0.0360	0.3230	0.3286	0.3646	0.6412	0.7269
		M	4	0.802	0.332	0.664	0.441	0.152	0.242	0.675	1.488	1.704
	80–89	F	4	0.763	0.485	0.970	0.940	0.245	0.254	0.295	1.740	2.217
		M	4	0.860	0.438	0.877	0.769	0.050	0.191	0.641	1.747	2.106
miR326	40–49	F	2	0.798	0.735	1.040	1.081	0.063	*	0.798	*	1.534
		M	1	0.17124	*	*	*	0.17124	*	0.17124	*	0.17124
	50–59	F	7	0.600	0.473	1.252	1.567	0.033	0.034	0.097	0.320	3.429
		M	1	0.77349	*	*	*	0.77349	*	0.77349	*	0.77349
	60–69	F	5	0.1291	0.0680	0.1521	0.0231	0.0121	0.0407	0.0740	0.2454	0.3957
		M	8	0.1028	0.0253	0.0715	0.0051	0.0186	0.0467	0.0809	0.1693	0.2126
	70–79	F	4	0.698	0.548	1.096	1.201	0.069	0.088	0.192	1.814	2.338
		M	4	0.300	0.127	0.253	0.064	0.048	0.069	0.280	0.551	0.593
	80–89	F	4	0.180	0.102	0.203	0.041	0.069	0.073	0.083	0.385	0.485
		M	4	0.156	0.124	0.247	0.061	0.006	0.008	0.046	0.412	0.524

## Data Availability

The data presented in this study are available on request from the corresponding author.
